# Supplementary material for: Identification of Freezing-Responsive microRNAs and Their Targets in Chinese Jujube by Small RNA and Degradome Sequencing
Source: Int J Mol Sci. 2026 Sep 4;27(17):7912. doi: 10.3390/ijms27177912 (PMC13566890; doi:10.3390/ijms27177912)
Supplement: Supplementary file 1 [file ijms-27-07912-s001.zip › Supplementary Material S2/novel_miR_41.pdf]

Provisional ID	:	novel_miR_41
Score total	:	25.6
Score for star read(s)	:	3.9
Score for read counts	:	23.6
Score for mfe	:	0.3
Score for randfold	:	-2.2
Score for cons. seed	:	
Total read count	:	58
Mature read count	:	57
Loop read count	:	0
Star read count	:	1

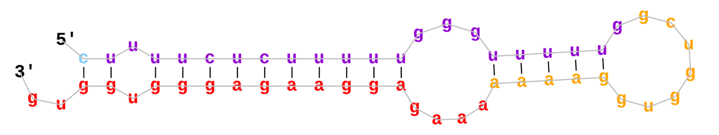

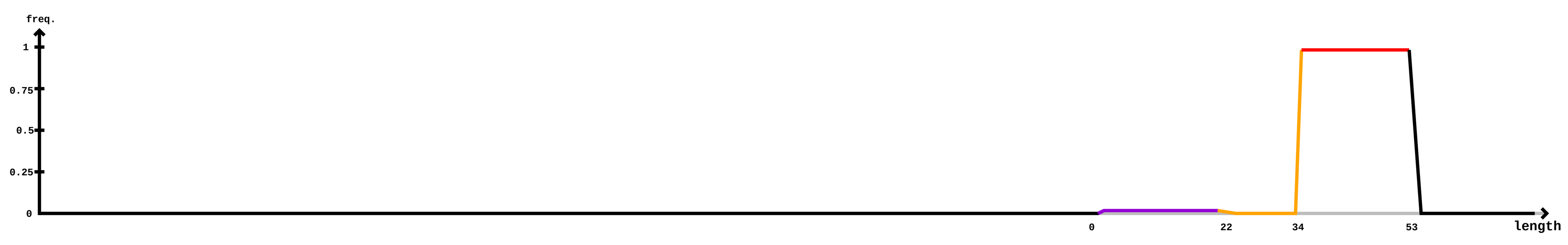

Star Mature

```
5'- ggcaacaugagugagcccauuggaagaacauuaggauuucgaugauuaggguuugaagaucugaaauuucuugauggauucauaucuaugaauguuuuggcuugcuucccuuaccacauuuuggcugugggagaaacuacccuuaaaauaguagagugggaaauggaaguuuguacuuuucucuuuuuggguuuuuggcugguggaaaaaaagaggaagaggguggugUaaaagaauccaagagcauuaa -3' obs
    ggcaacaugagugagcccauuggaagaacauuaggauuucgaugauuaggguuugaagaucugaaauuucuugauggauucauaucuaugaauguuuuggcuugcuucccuuaccacauuuuggcugugggagaaacuacccuuaaaauaguagagugggaaauggaaguuuguacuuuucucuuuuuggguuuuuggcugguggaaaaaaagaggaagaggguggugUaaaagaauccaagagcauuaa     exp
    .....((((((((((.(((((..(((((..(((((.((((((..........)))))).)))))...)))))))))).))))).)).)))((((((((((....(((((.((.((.(((((((.((((((((........))))...))))))))))))).)).)))))((((((((.((((((((((...(((((........)))))....))))))))))).)))))))).....)).))))))))..   reads   mm   sample
    ....................................................................................................................................................................uuuucuUuuuuuggguuuuug..........................................................   1   1   LT-1
    ....................................................................................................................................................................................................aaagaggaagaggguggug......................   26   0   LCK-2
    ....................................................................................................................................................................................................aaagaggaagaggguggug......................   30   0   LCK-1
    ....................................................................................................................................................................................................aaagaggaagagggNggug......................   1   1   LCK-1
```
